# Identification of Cross-Protective Potential Antigens against Pathogenic *Brucella* spp. through Combining Pan-Genome Analysis with Reverse Vaccinology

**DOI:** 10.1155/2018/1474517

**Published:** 2018-12-09

**Authors:** Yasmin Hisham, Yaqoub Ashhab

**Affiliations:** Palestine-Korea Biotechnology Center, Palestine Polytechnic University, Hebron, State of Palestine

## Abstract

Brucellosis is a zoonotic infectious disease caused by bacteria of the genus *Brucella*. *Brucella melitensis*, *Brucella abortus*, and *Brucella suis* are the most pathogenic species of this genus causing the majority of human and domestic animal brucellosis. There is a need to develop a safe and potent subunit vaccine to overcome the serious drawbacks of the live attenuated *Brucella* vaccines. The aim of this work was to discover antigen candidates conserved among the three pathogenic species. In this study, we employed a reverse vaccinology strategy to compute the core proteome of 90 completed genomes: 55 *B. melitensis*, 17 *B. abortus*, and 18 *B. suis*. The core proteome was analyzed by a metasubcellular localization prediction pipeline to identify surface-associated proteins. The identified proteins were thoroughly analyzed using various *in silico* tools to obtain the most potential protective antigens. The number of core proteins obtained from analyzing the 90 proteomes was 1939 proteins. The surface-associated proteins were 177. The number of potential antigens was 87; those with adhesion score ≥ 0.5 were considered antigen with “high potential,” while those with a score of 0.4–0.5 were considered antigens with “intermediate potential.” According to a cumulative score derived from protein antigenicity, density of MHC-I and MHC-II epitopes, MHC allele coverage, and B-cell epitope density scores, a final list of 34 potential antigens was obtained. Remarkably, most of the 34 proteins are associated with bacterial adhesion, invasion, evasion, and adaptation to the hostile intracellular environment of macrophages which is adjusted to deprive *Brucella* of required nutrients. Our results provide a manageable list of potential protective antigens for developing a potent vaccine against brucellosis. Moreover, our elaborated analysis can provide further insights into novel *Brucella* virulence factors. Our next step is to test some of these antigens using an appropriate antigen delivery system.

## 1. Introduction

Brucellosis is a global zoonotic infectious disease caused by bacteria of the genus *Brucella*. The disease is a serious public health threat worldwide, particularly in the developing countries of Central Asia, Africa, South America, and the Mediterranean region [1]. Brucellosis affects mammals, causing abortion and infertility in affected animals. Infection can spread from animals to humans mainly via ingestion of unpasteurized milk or dairy products and, to a lesser extent, via direct contact with infected animals [2]. In humans, brucellosis can cause a severe febrile disease with various clinical complications ranging from mild to severe symptoms including undulant fever, joint pain arthritis, endocarditis, and meningitis [3–5]. *Brucella* is a genus of Gram-negative facultative intracellular bacteria that belongs to the class Alphaproteobacteria. Currently, the genus consists of 10 species that are classified based on their host preferences [6]. Although several *Brucella* species are potentially zoonotic agents, *Brucella melitensis* (*B. melitensis*), *Brucella abortus* (*B. abortus*), and *Brucella suis* (*B. suis*) are considered the most pathogenic *Brucella* species that have a serious impact on public health and the livestock industry [7, 8].

The strategy used to control brucellosis depends mainly on the massive vaccination of domestic animals to prevent disease spread to healthy animals and to humans. Typically, after achieving a very low prevalence rate in domestic animals (below 1%), a strict surveillance strategy can be applied to get rid of infected animals [9, 10]. Currently, there are only a few vaccines that are used to control brucellosis in animals such as *B. abortus* strains S19 and RB51, *B. melitensis* strains Rev.1 and M5, and *B. suis* strain S2 [11]. Almost all these vaccines are live attenuated strains derived by *in vitro* serial passages from field strains. Despite their extensive global use, these live attenuated vaccines suffer from various drawbacks, such as pathogenicity to humans and residual virulence in animals, which can cause abortion, orchitis, and infertility [12, 13]. Moreover, it is difficult to differentiate infected animals from vaccinated animals by serological tests. These drawbacks have prompted several research groups to attempt the development of safer subunit vaccines. Two conditions are essential to design a good subunit vaccine: first is the selection of appropriate protective antigens, and second is the selection of a safe and efficient vehicle to deliver these antigens to evoke a protective immune response.

During the last two decades, a number of *Brucella* antigens have been identified, such as Omp16, Omp19, Omp25, Omp31, SurA, Dnak, trigger factor (TF), ribosomal protein L7L12, bacterioferritin (BFR) P39, and lumazine synthase BLS [14–21]. These antigens were selected based on empirical screening approaches that are typically laborious and expensive and require strict safety precautions and particular lab facilities, as the relevant species of *Brucella* are classified as biosafety level 3 microorganisms. This insufficiency of the empirical methods represents a great need for a rational and comprehensive approach to discover potential antigen candidates that can be used to develop a safe and effective anti *Brucella* vaccine.

In contrast to the conventional vaccine development approaches that require cultivation and extensive empirical screening, the reverse vaccinology (RV) approach is an interesting *in silico* approach to identify protective antigens using pathogen genomic data. The method was first developed by Rappuoli and Pizza et al. to discover protective antigens of serogroup B meningococcus [22, 23]. Since then, RV has been implemented to identify protective antigens of numerous pathogens [24, 25]. Two studies have applied RV to identify *Brucella* antigens [26, 27]. A major limitation of these studies is that they performed RV analysis using only one strain, namely, *B. melitensis* 16M. Moreover, they employed inadequate antigen selection criteria. Due to the interstrain gene content diversity, it has become crucial to analyze several strains of a given bacterial species or genus to identify the core genome that contains the desired universal protective antigens [28].

In this study, we aimed to discover potential antigen candidates that are conserved among *B. melitensis*, *B. abortus*, and *B. suis*, which are the *Brucella* species associated with human and domestic animal disease. Our RV approach is an improved version based on determining the core genes of an extensive number of genomes from the three aforementioned *Brucella* species, followed by a rational antigen selection strategy. To our knowledge, this is the first study to combine pan-genome and reverse vaccinology approaches to identify potential protective antigen that can be used to develop a universal vaccine against the three most pathogenic *Brucella* species.

## 2. Materials and Methods

**Figure 1:**
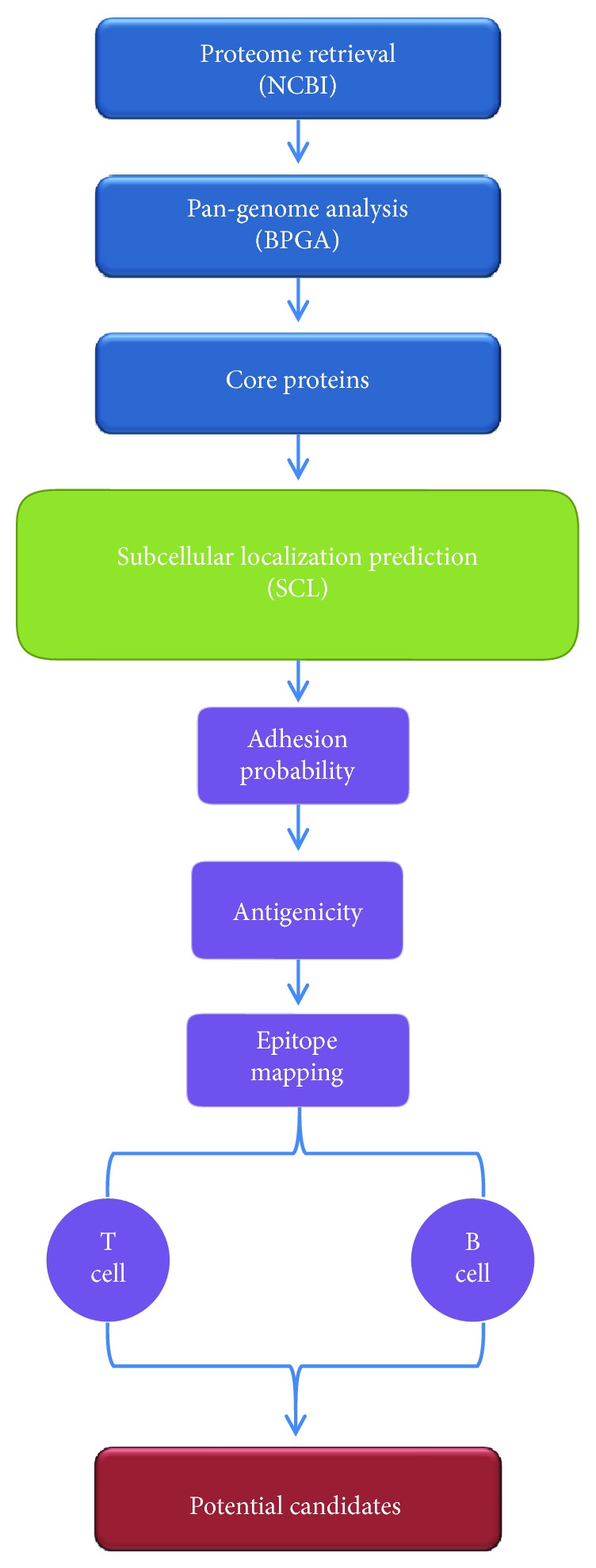
A schematic flow diagram of the reverse vaccinology protocol applied in this study to select potential vaccine candidates of the three *Brucella* species.

Our *in silico* antigen prediction protocol is depicted in Figure 1. In the first phase, the retrieved proteomes were analyzed to extract the core proteome (the set of homologous proteins that are present in all analyzed strains of the three *Brucella* species). The identified core proteome is subsequently analyzed using a subcellular localization prediction pipeline to identify outer membrane and periplasmic proteins. In the last stage, we employed various rigorous filters to prioritize proteins based on features that are strongly associated with protective antigenicity, including adhesion, overall protein antigenicity, and density of B cell and T-cell epitopes. Unless otherwise specified, the default parameters were used for all prediction tools.

### 2.1. Data Retrieval

The full multi-FASTA format protein sequences of 55 *B. melitensis*, 17 *B. abortus*, and 18 *B. suis* genomes were downloaded from the Microbial Genomes Resources-NCBI (https://www.ncbi.nlm.nih.gov/genome) (as of March 2018). Accession numbers, strain names, and number of proteins are shown in Supplementary File 1.

### 2.2. Pan-Genome Analysis

In order to identify the core proteins, the 90 proteomes were analyzed by the Bacterial Pan-Genome Analysis (BPGA) tool using the default parameters [29]. In the input preparation for clustering step, option number 4 (use any protein FASTA files) was chosen. To ensure fast and accurate clustering, BPGA uses USEARCH as a default protein clustering tool with an identity cut off = 50%.

### 2.3. Subcellular Localization (SCL)

**Figure 2:**
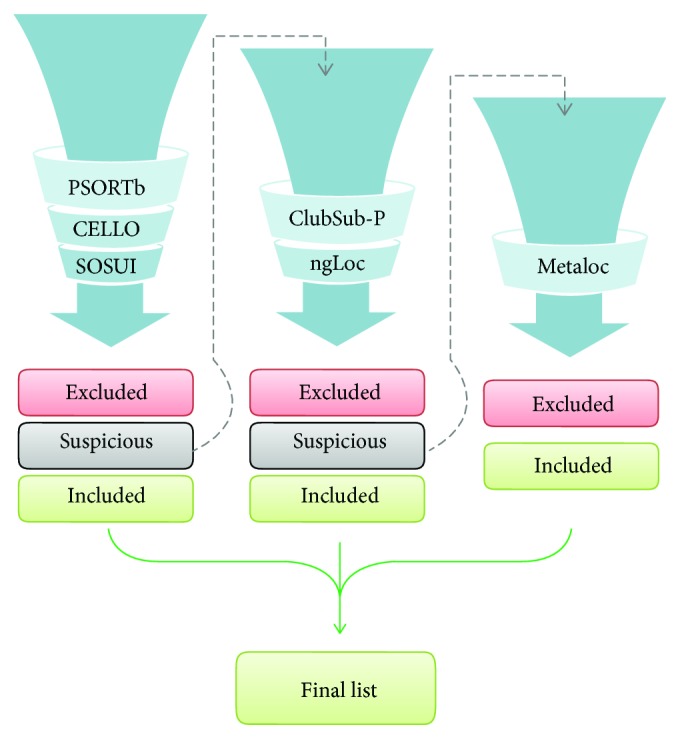
General workflow of our subcellular localization prediction pipeline. A total of 6 tools were applied to the core proteins (1939 proteins) that resulted from pan-genome analysis. The process starts with first group of tools consisting of PSORTb, CELLO, and SOSUI. The proteins with uncertain prediction have to move to the second phase to be analyzed by another two tools, namely, ClubSub-P and ngLoc. The uncertain proteins resulting from the second phase are subjected to the final prediction tool MetaLoc.

Next, the core proteome was analyzed to predict outer membrane and periplasmic proteins. In this step, a previously developed homemade pipeline for SCL prediction was performed (Y. Ashhab, unpublished data). The pipeline employs different SCL prediction tools in three phases of positive and negative selections (Figure 2). Positive selection was performed for outer membrane (OM) and/or periplasmic (P) proteins. Negative selection was performed for inner membrane (IM), cytoplasmic (CYT), and extracellular (EX) proteins.

The three tools used in the first phase were as follows: PSORTb v3.0.2, CELLO v.2.5, and SOSUI-GramN [30–32]. In this stage, the positive selection was implemented for proteins that were predicted as OM or P by at least two of the three tools and were therefore included. Negative selection was implemented for proteins that were predicted as IM, EX, or CYT by at least two of the three tools and were therefore excluded. Proteins that were predicted with “unknown” subcellular location by at least one of the three tools and OM and/or P by one of the three tools were considered uncertain proteins and were subjected to the second phase of selection. The two tools used in the second phase of selection were as follows: ClubSub-P and ngLoc [33, 34]. Again, resulting proteins were divided into three categories. Positive selection was implemented for proteins that were predicted as OM or P by at least one of the two tools and were therefore included. Negative selection was implemented for proteins that were predicted as IM, EX, or CYT by at least one of the two tools and were therefore excluded. Proteins predicted with “unknown” subcellular location by one of the two tools were defined as uncertain. These uncertain proteins were subjected to a third phase of selection with the metaprediction tool, MetaLoc [35]. Proteins in this final step were divided into two categories: included for OM and P or excluded for the other sites. Included proteins from the three phases were collected for further analysis.

### 2.4. Adhesion Probability

Adhesion probability of the surface-associated proteins that summed up from the SCL prediction was predicted by Vaxign tool [36]. Proteins with an adhesion score higher than 0.5 were selected for further analysis.

### 2.5. Protein Antigenicity

Antigenicity of surface-associated proteins was predicted using two tools: AntigenPro which computed antigenicity based on amino acid sequence features [37] and VaxiJen which computed antigenicity based on physicochemical properties of amino acid sequence [38].

### 2.6. T-Cell Epitope Prediction

Surface-associated proteins were also subjected to sequential epitope mapping in order to indicate their ability to bind to immune cells. T-cell epitopes were predicted for major histocompatibility complex (MHC) class I and class II, and the number of potential binding alleles for each protein was determined. ProPred1, and ProPred were used for MHC class I and MHC class II epitopes, respectively [39, 40]. The epitope density in a given protein was calculated for each class of MHC by dividing the number of predicted epitopes over the length of that given protein. In addition, epitope coverage was calculated by dividing the number of alleles with positive predictions over the total number of analyzed alleles.

### 2.7. B-Cell Epitope Prediction

BCPred and AAPred were used for B-cell epitope prediction [41, 42]. Using the default parameters, epitopes with a score ≥ 0.8 were accepted. The density of the B-cell epitope for a given protein was calculated by dividing the number of predicted B-cell epitopes over the protein length.

### 2.8. Prioritization of Protective Antigens

In this step, a cumulative score for the proteins with adhesion score ≥ 0.5 was calculated using the prediction scores of protein antigenicity, MHC-I and MHC-II epitope densities, allele coverage for both classes of MHC, and B-cell epitope density. The score for each feature was normalized to “1” as the highest possible value and “0” as the lowest possible value. The protein antigenicity score was the average of the two tools: VaxiJen score and AntigenPro score. The B-cell epitope density score was the average density of the two tools: AAPred and BCPred.

### 2.9. Exclusion of Dubious Proteins

Proteins that show significant homology to host proteins or proteins that have low molecular weight were excluded from the final list. To remove proteins with significant homology to host protein sequences, the selected antigens were subjected to homology search against proteomes using BLASTp tool at https://blast.ncbi.nlm.nih.gov with the following parameters: database: reference proteins (refseq_protein); organisms: human, sheep, goat, cattle, and pig; and *E*-value cutoff: 0.001. Antigens that show ≥35% identity to any host protein were excluded. Molecular weight of small proteins was estimated using ExPASy tool [43]. Proteins having a molecular weight of <10 kDa were excluded.

### 2.10. Protein Annotation and Domain Search

In addition to the one-line annotation description provided by NCBI, we performed a thorough manual annotation to determine the most likely biological function assigned to the selected antigens. For this purpose, we used the following protein annotation servers: Blannotator, Pannzer, and eggNOG [44–46]. Furthermore, the conserved domain search was predicted using BLAST CD-search tool. BOCTOPUS 2 was used to predict the topology of transmembrane beta-barrel proteins [47].

## 3. Results

**Figure 3:**
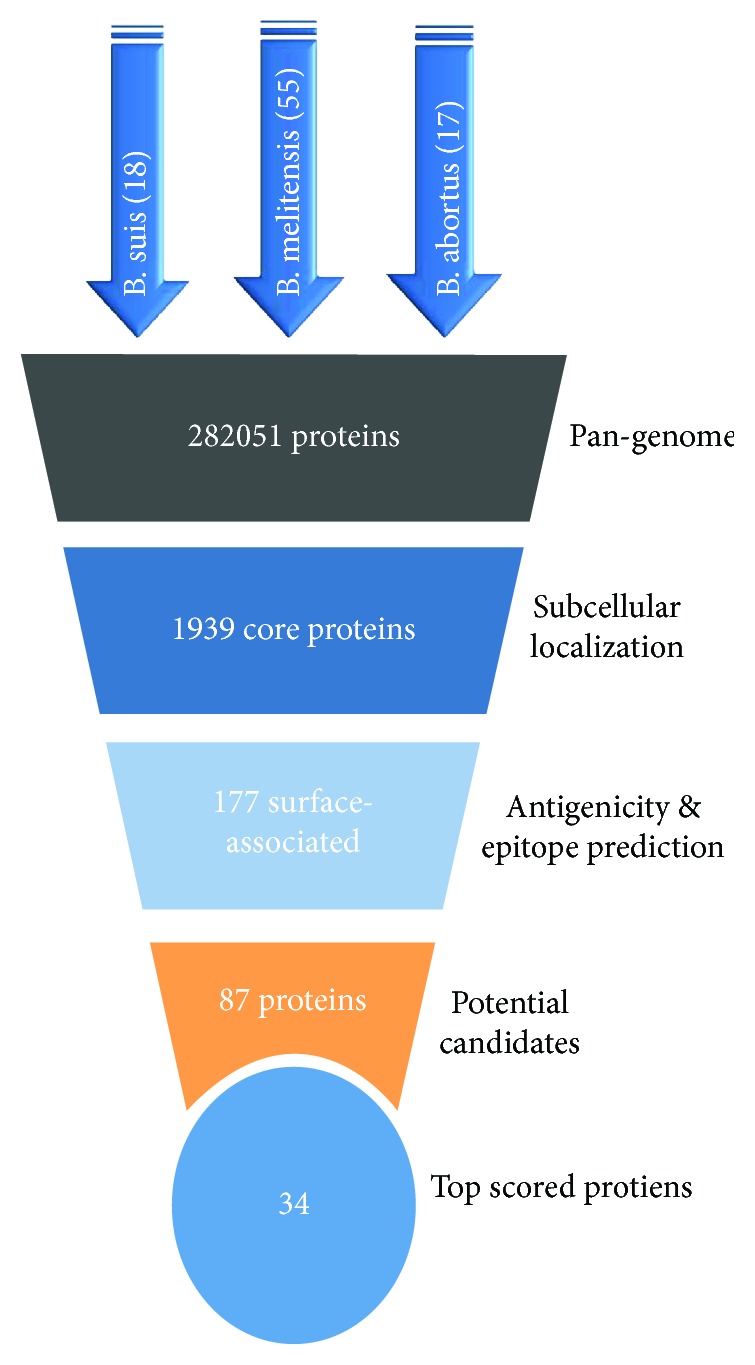
Summary of the resultant proteins in each step of our vaccine candidate selection. The total number of analyzed proteins was 282051, the pan-genome analysis resulted in 1939 core proteins. Next, the SCL prediction pipeline resulted in 177 proteins. The number of potential protein candidates was 87, and finally the antigens with the top cumulative antigenicity score were 34.

Results of our reverse vaccinology analysis to identify potential antigen candidates that can be used to develop a universal vaccine against *Brucella* are summarized in Figure 3.

### 3.1. Pan-Genome Analysis

**Figure 4:**
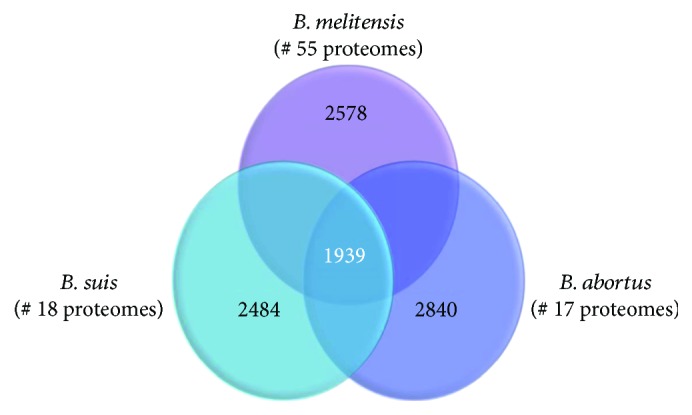
This Venn diagram shows the results of the pan-genome analysis of the three *Brucella* species. The numbers of genomes for each species are indicated. The number of core proteins for each species is shown in each corresponding circle, while the number of core proteins common for all the three species is shown in the intersection area.

Core proteins were initially identified for each species alone then for the three species together. The number of core proteins for the 17 strains of *B. abortus* was 2840, while for the 55 strains of *B. melitensis* was 2578, and for the 18 strains of *B. suis* was 2484. The number of core proteins for all 90 proteomes of the three species was 1939.  Figure 4 shows a Venn diagram of core proteins for the three species.

### 3.2. Subcellular Localization (SCL)

From the 1939 core proteins, the surface-associated proteins were selected by our SCL prediction pipeline as shown in Figure 2. In the first phase, 151 proteins were included, 1639 were excluded, and 149 were labeled uncertain. These 149 proteins were subjected to the second phase of analysis in the pipeline, which excluded 104 proteins and included 16 proteins. The rest 29 uncertain proteins were subjected to the final phase of analysis. Of these 29 proteins, 19 were excluded, and 10 were included. Thus, the total number of proteins included from the three phases was 177 proteins, making up the final list of surface-associated proteins (see Supplementary File 2).

### 3.3. Prioritization of Protective Antigens

As adhesion capacity was shown to be a key feature common to many experimentally verified protective antigens [48], we decide to use adhesion scores, produced by Vaxign, to scale the 177 surface-associated proteins in a descending order. The proteins with an adhesion score ≥ 0.5 (38 proteins) were considered antigens with “high potential,” while those with an adhesion score between 0.4 and 0.5 are considered antigens with “intermediate potential” (see Supplementary File 3). The 38 proteins with high potential were ranked based on a cumulative score that was derived from protein antigenicity, density of MHC-I and MHC-II epitopes, MHC allele coverage, and B-cell epitope density scores (Table 1). For the detailed score calculation, see Supplementary File 4. Of these 38 high-potential proteins, cytochrome c was excluded to avoid autoimmune response because of its homology to host proteins. In addition, 3 proteins with low molecular weight (6.7 kDa, 7.9 kDa, and 9.4 kDa) were excluded because proteins with a molecular weight < 10 kDa are poorly immunogenic [49].

Among the 34 proteins classified as antigens with “high potential,” 15 were annotated as hypothetical or unknown function. To gain more insight into the biological functions of these proteins, the 34 proteins were manually annotated using various protein annotation and conserved domain searching tools. The number of proteins with unknown function decreased from 15 to 4 (Table 1). Our domain analysis showed that LomR is a frequently found domain among the antigens with high potential. This domain is a classical domain associated with many outer membrane proteins with transmembrane *β*-barrel scaffold that belongs to Gram-negative porin superfamily. The results of protein annotation were analyzed to identify any biological pattern that may be associated to the predicted antigens. Although there are little resources to investigate gene ontology of *Brucella* proteins, the 34 high-potential antigens tend to be associated with certain biological processes, including transmembrane transport (especially ions, iron, and small organic nutrients), membrane assembly, cell adhesion, and pathogenesis (Table 1).

## 4. Discussion

Brucellosis is a global zoonotic infection with a devastating economic impact on livestock sector and public health in many developing countries [50]. There is an unmet need to develop safe and efficient vaccine to fight brucellosis. This need was addressed in 2017 by launching a global prize competition of 30 million US dollars for developing a safe and efficient vaccine against Brucellosis (https://brucellosisvaccine.org). The first step in developing such a vaccine would be to determine the protective antigens of these bacteria. Therefore, the aim of this study was to determine a set of universal and protective antigens that can be used to develop a vaccine against the three most pathogenic species of *Brucella* (*B. melitensis*, *B. abortus*, and *B. suis*) that are responsible for most cases of brucellosis among domestic animals and humans. We have combined a pan-genome analysis with rational selection steps of reverse vaccinology to determine a manageable shortlist of *Brucella* antigens. We identified 34 potential cross-protective antigens from 90 complete proteomes covering the three species.

Although two recent studies have published their pan-genome analysis results of *Brucella* [51, 52], we decided to perform our own pan-genome analysis because these two studies were performed with a relatively limited number of genomes to study the variation and relatedness among almost all species of *Brucella*, while our objective was to identify the core genome for *B. melitensis*, *B. abortus*, and *B. suis*.

A critical factor in applying a successful RV approach is to have a good understanding of the natural immune response to the pathogen of interest. In the case of *Brucella* infection, immunity is achieved by triggering both cellular and humoral mechanisms. Cell-mediated immunity plays a critical role in protection against these intracellular bacteria, and it is mainly mediated by Th1 response [53]. On the other hand, passive immunization of animals with antibodies from immunized animals provides protection against *Brucella* infection [54–56]. Several studies have shown that surface-associated antigens of Gram-negative bacteria are essential to confer not only protective humoral immunity but also cell-mediated immunity against intracellular bacteria [57–59]. Therefore, our first RV filter was to identify outer membrane and periplasmic proteins of *Brucella*. Instead of using a single tool to identify these surface-associated proteins, we used a home-made pipeline which outperforms the currently available SCL prediction tools (Y. Ashhab, unpublished data). Our pipeline minimizes the possibility of excluding proteins that are assigned with unknown SCL, a scenario common to all SCL prediction tools.

In addition to surface-associated localization, we endeavor to use a feature that is strongly associated to protective immune response. Ong et al. investigated a large group of protective bacterial antigens to reveal the most prominent biological features shared among these proteins. They found that the two most important features shared among protective antigens of Gram-negative bacteria are adhesion and association with cell surface [48]. Consequently, after predicting the list of surface-associated proteins (177 proteins), adhesion capability was predicted and used to rank these proteins.

It has been proven that proteins with high epitope density have significantly greater immunogenicity [60, 61]. Accordingly, proteins with high density of predicted epitopes are more potential vaccine candidates. Despite the growing numbers of immunobioinformatic tools that can predict MHC class I- and class II-binding peptides, these tools are almost exclusive to human and mouse MHC alleles. Unfortunately, domestic animals, such as sheep, goats, and cows, have limited MHC epitope data and prediction tools. However, we noticed a good agreement between the epitope prediction results of human and cow MHC alleles using ProPred server (see Supplementary File 3). This similar binding behavior would support the validity of our MHC scoring and its contribution to enhance the selection of universal antigens.

We have examined the virulence and pathogenicity of our protein list using VirulentPred, a virulence prediction tool [62], and MP3, a metapathogenicity prediction tool [63], respectively. However, the results of these two tools were not informative to rank the antigens; the majority of the 177 surface-associated proteins gave a positive prediction. Therefore, we decided to exclude these two tools.

In this study, we provide a rational reverse vaccinology approach against the three most clinically important *Brucella* species. Two previous studies have employed reverse vaccinology to identify antigens of *B. melitensis* strain 16M [26, 27]. However, these studies suffered from a number of limitations. The major limitation is that they were restricted to one genome and therefore their results cannot be extrapolated either to different strains of *B. melitensis* or to the different pathogenic species of *Brucella*. Although the two studies were performed on the same strain of *B. melitensis*, they have no overlapping in the final list of selected antigens.

In this study, 34 proteins were identified as potential protective antigens that can serve to develop a novel universal vaccine against brucellosis. As 15 of these proteins have been deposited in GenBank without assigned function (11 hypothetical proteins and 4 proteins containing domains of unknown function (DUF)), we decided to perform a thorough *in silico* analysis to gain more insight on the function of all the 34 proteins. As shown in Table 1, the potential antigens tend to fall into a few categories of biological functions. An interesting protein family under these categories is the outer membrane proteins (OMPs) that possess 8–10 strands of *β* sheet. Of the 34 proteins, 8 belong to this subfamily of OMPs. Despite their involvement in the transport of small solutes, it was found that small-size OMPs (8–10 *β* sheet strands) tend to have a key role in adhesion, invasion, and evasion to contribute to the tissue damage and bacterial spread across tissue barriers [64]. Indeed, most of the shortlisted OMPs such as Omp19, Omp25, Omp31, OmpA, and OmpW are associated with *Brucella* virulence and some of them showed a significant level of immune response when used as subunit vaccines [65–71].

A second interesting group of proteins is related to iron acquisition, including the hypothetical protein “WP_002966226.1,” TonB-dependent receptor “WP_004691650.1,” heme transporter BhuA “WP_023080793.1,” and the iron ABC transporter substrate-binding protein “WP_004681306.1.” The importance of iron for survival and virulence of *Brucella* is well documented, and targeting proteins essential for iron acquisition is a promising strategy to develop effective bacterial vaccines [72].

A third group of proteins is the ABC transporters. This family of transporters is essential to secure uptake of various vital nutrients that cannot be produced by *Brucella*. It is believed that the ABC transporter proteins play a role in *Brucella* survival within the host during its infectious life cycle [73]. Furthermore, it has been reported that the ABC proteins are able to induce immunity, making them potential vaccine targets [74, 75].

An interesting identified candidate is VirB1, which is a component of the type IV secretion system (T4SS) of *Brucella* spp. This secretion system in *Brucella* is a well-known virulence factor, which is responsible for survival, intracellular trafficking, and replication of *Brucella* inside the infected host cells [76–78]. Using our selection approach, we were able to identify some potential antigens that are periplasmic proteins with critical roles in outer membrane biogenesis and integrity. Among these proteins are BamD and BamE, which are critical components of the *β*-barrel assembly machinery (BAM) [79]. Another interesting protein is the LPS-assembly protein LptD that is an essential component of the lipopolysaccharide transport (Lpt) machinery [80]. It is plausible that targeting one of these essential outer membrane biogenesis machineries would have a severe effect on bacterial survival.

Among the list of potential antigens, two proteins belong to the BA14K immunoreactive protein family, which is a poorly characterized group of surface antigens. It has been reported that this family can strongly induce both cellular and humoral immune responses [81, 82]. Further investigation is needed to understand the functions of these two factors and their potential as protective antigens.

As our aim was to identify universal antigens conserved among the three pathogenic species (*B. melitensis*, *B. abortus*, and *B. suis*), it is possible that our approach could have missed some interesting species-specific antigens. Although we ranked the 177 surface-associated proteins using adhesion, which is a crucial biological property strongly associated with a significant number of experimentally verified protective antigens, we cannot exclude the possibility that some potential antigens are missed from our “high-potential” 34 antigens. In fact, a few interesting candidates were ranked in the “intermediate-potential” antigens (see Supplementary File 3). Among these interesting candidates are Bp26 and SOD. Bp26, or immunoreactive Omp28, is an antigen protein that is widely described as a potential vaccine candidate [27, 70, 83, 84]. In addition, it has been found to be immunogenic in both goats and humans and it provides a significant protection rate in BALB/c mice [84, 85]. Superoxide dismutase (SOD) proteins have been reported in *B. abortus* and found to be responsible for host macrophage bursts. Thus, it is considered a promising antigen [86]. This antigen has also been found in *B. melitensis* as an immunodominant protein [87]. Moreover, SOD is considered a potential antigen with promising protective properties [70, 88, 89]. Here, we were able to identify two superoxide dismutases, namely, SOD_Cu-Zn and SOD_Mn within the list of “intermediate-potential” antigens.

It is worth to mention that our extended list of antigens, either with high and/or with intermediate potential, does not contain various cytoplasmic proteins that were previously suggested as possible antigens [15–17]. Among these antigens, lumazine synthase BLS is the most interesting candidate because it showed a good humoral and cell-mediated response and it induces protective immunity in mice [15].

## 5. Conclusion

Bioinformatics is a strong approach for vaccine candidate discovery as it offers a faster, cheaper, and safer method to identify potential vaccine targets when compared with traditional laboratory identification methods, particularly when dealing with risk group 3 microorganisms such as *Brucella*. Here, we provide a RV strategy that combines pan-genome analysis with a meta-SCL pipeline, followed by a rational-based selection that can rank surface-associated antigens according to their potential protective immunogenicity. Using our approach, we were able to identify several potential cross-protective candidates. The majority of the top-ranked antigens are strongly associated to bacterial virulence, and, therefore, it is plausible to assume that some of these antigens can form a solid base to design an efficient and safe vaccine against animal and human brucellosis. Further experiments are needed to test immunogenicity and protection level of these proteins.

## Figures and Tables

**Table 1:** High-potential protein list, with their adhesion score, cumulative results, and consensus annotation resulting from Blannotator, Pannzer, and eggNOG tools. The shown biological function is extracted from protein family databases as well as the indicated literature in the last column.

Protein ID (NCBI)	Length (aa)	Single-line annotation (NCBI)	Adhesion score	Cumulative score of 5	Annotation note (by Blannotator, Pannzer, and eggNOG)	Domains (CD-search)	No. of *β* sheet strands	Biological functions	Reference
WP_004684144.1	274	Porin family protein	0.59	4.56	Porin opacity type (Pannzer), heat-resistant agglutinin 1 (eggNOG)	LomR	8	Small solute transport, colonization, and adhesion	[64, 90]
WP_002964666.1	227	OmpW family protein	0.55	4.52	OmpW family outer membrane protein (Pannzer, eggNOG), uncharacterized outer membrane protein y4mB (Blannotator)	OmpW	8	Stress response, small solute transport, and bacterial colonization	[64, 69, 91]
WP_002969562.1	155	Hypothetical protein	0.54	4.5	Not determined	No domain hits		ND	
WP_002966849.1	280	DUF1849 domain-containing protein	0.6	4.5	ATP/GTP-binding site domain-containing protein A (Pannzer), DUF1849 domain-containing protein (eggNOG)	DUF1849		Uptake of organic nutrient	
WP_002964611.1	351	DUF1176 domain-containing protein	0.54	4.44	DUF1176 domain-containing protein (eggNOG)	DUF1176		ND	
WP_004690357.1	284	Porin family protein	0.55	4.42	Heat-resistant agglutinin 1 (Pannzer, eggNOG), uncharacterized protein BRA0921/BS1330_II0913 (Blannotator)	LomR	8	Small solute transport, colonization, and adhesion	[64, 90]
WP_004681227.1	238	Type IV secretion system protein VirB1	0.66	4.38	Type IV secretion system protein VirB 1 (Pannzer, Blannotator), conjugal transfer protein (eggNOG)	Lysozyme-like superfamily		Adaptation to intracellular environment	[76, 92]
WP_002966226.1	182	Hypothetical protein	0.54	4.38	UPF0423 protein BAB2_0840 (Blannotator), pathogen-specific membrane antigen (Pannzer), periplasmic protein (eggNOG)	Tpd iron transport		Iron acquisition and virulence	[93, 94]
WP_002963597.1	121	Hypothetical protein	0.5	4.37	Membrane-bound lysozyme inhibitor of C-type lysozyme (by Blannotator, Pannzer, and eggNOG)	MliC		Immune evasion and colonization/virulence factor	[95]
WP_004688070.1	192	Hypothetical protein	0.59	4.32	Not determined	No domain hit		ND	
WP_002966502.1	126	Hypothetical protein	0.62	4.32	Outer membrane lipoprotein omp10 (by Blannotator, Pannzer, and eggNOG)	No domain hit		Virulence	[65]
WP_002964322.1	329	Hypothetical protein	0.59	4.23	31 kDa transporter (Blannotator), alkanesulfonate transporter substrate-binding subunit (Pannzer), trap transporter solute receptor taxi family (eggNOG)	TRAP_TAXI		Nutrient transport, pathogenicity, and colonization	[96]
WP_002971090.1	267	Hypothetical protein	0.54	4.22	Outer membrane beta-barrel domain protein (Pannzer)	OM_channel superfamily	10	Adhesion	[64, 97]
WP_004691650.1	620	TonB-dependent receptor	0.53	4.21	Iron compound TonB-dependent receptor (Pannzer), involved in the active translocation of vitamin B12 (cyanocobalamin) across the outer membrane to the periplasmic space. It derives its energy for transport by interacting with the transperiplasmic membrane protein TonB (by similarity) (eggNOG)	BtuB		Iron acquisition and vitamin B12 transport	[93, 94, 98]
WP_002971481.1	168	Outer membrane protein assembly factor BamE	0.69	4.21	Outer membrane protein assembly factor BamE (Pannzer), smpa omla domain-containing protein (eggNOG)	BamE		Cell envelope biogenesis and OMP assembly	[99]
WP_004683739.1	236	Porin family protein	0.56	4.21	Autotransporter outer membrane beta-barrel domain-containing protein fragment (Pannzer), hemin-binding protein (eggNOG)	LomR	8	Iron acquisition	[64, 90]
WP_004691134.1	403	Hypothetical protein	0.62	4.15	Putative L,D-transpeptidase YafK (Blannotator), pollen allergen Poa pIX/Phl pVI (Pannzer), ErfK ybiS ycfS ynhG family protein (eggNOG)	Yafk		Envelope biogenesis and stress response	[100]
WP_002965482.1	439	Sugar ABC transporter substrate-binding protein	0.55	4.11	ABC-type sugar transport system periplasmic component (Pannzer), extracellular solute-binding protein family 1 (eggNOG)	PBP2_TMBP_like		Uptake of organic nutrient and invasion/virulence	[73]
WP_002964333.1	220	OmpA family protein	0.56	4.09	Probable lipoprotein YiaD (Blannotator), cell envelope biogenesis protein OmpA (Pannzer), OmpA motb domain protein (eggNOG)	OmpA		Cell envelope biogenesis, adhesion, invasion/intracellular survival, and evasion of host defense	[67]
WP_023080793.1	661	Heme transporter BhuA	0.51	4.05	Heme transporter BhuA (Blannotator, Pannzer), receptor (eggNOG)	CirA superfamily		Iron acquisition, virulence, and association for bacterial persistence	[93, 94, 101]
WP_002964719.1	261	Porin family protein	0.57	4.04	31 kDa outer membrane immunogenic protein (Omp31) (by Blannotator, Pannzer, and eggNOG)	LomR	8	Hemin-binding proteins and virulence	[64, 102, 103]
WP_002966352.1	156	DUF2271 domain-containing protein	0.7	4.02	Tat pathway signal protein (Pannzer), predicted periplasmic protein (DUF2271) (eggNOG)	DUF2271		ND	
WP_004690579.1	429	Cell wall hydrolase	0.51	4	Cell wall hydrolase (Pannzer, eggNOG)	CwlJ		Cell envelope biogenesis	[104]
WP_004683944.1	212	Porin family protein	0.62	3.98	Omp 25 (Pannzer), membrane (eggNOG)	LomR	8	Virulence and adhesion	[64, 97, 105]
WP_011068938.1	792	LPS-assembly protein LptD	0.5	3.93	LPS-assembly protein LptD (Pannzer), involved in the assembly of LPS in the outer leaflet of the outer membrane. Determines N-hexane tolerance and is involved in outer membrane permeability. Essential for envelope biogenesis (by similarity) (eggNOG)	LptD		Cell envelope biogenesis	[106]
WP_002967296.1	166	BA14K family protein	0.6	3.92	Immunoreactive BA14K (Pannzer, eggNOG)	BA14K		Lectin-like activity and virulence	[81, 82]
WP_002964622.1	170	BA14K family protein	0.58	3.92	Glutelin (Pannzer), BA14K (eggNOG)	BA14K		Lectin-like activity and virulence	[81, 82]
WP_004683466.1	213	Membrane protein	0.55	3.9	25 kDa outer membrane immunogenic protein Omp 25 (Blannotator, Pannzer), membrane (eggNOG)	LomR	8	Virulence and adhesion	[64, 97, 105]
WP_002963776.1	115	DUF2147	0.6	3.89	sn-Glycerol-3-phosphate ABC transporter ATP-binding protein (Pannzer), uncharacterized protein conserved in bacteria (DUF2147) (eggNOG)	COG4731		Nutrient transport and invasion/virulence	[73]
WP_004681306.1	367	Iron ABC transporter substrate-binding protein	0.51	3.88	Periplasmic binding ABC transporter (Pannzer), solute-binding protein (eggNOG)	AfuA		Iron acquisition and invasion/virulence	[73]
WP_002964998.1	177	Hypothetical protein	0.67	3.72	Outer membrane lipoprotein omp19 (by Blannotator, Pannzer, and eggNOG)	Inh		Protease inhibitor and alters the outer membrane properties	[65, 107]
WP_002964530.1	287	Outer membrane protein assembly factor BamD	0.52	3.69	Outer membrane protein assembly factor BamD (Blannotator, Pannzer), part of the outer membrane protein assembly complex, which is involved in assembly and insertion of beta-barrel proteins into the outer membrane (eggNOG)	BamD		Cell envelope biogenesis, OMP assembly, and required for bacterial viability	[99]
WP_006278325.1	261	Hypothetical protein	0.5	3.69	Proline-rich region: proline-rich extensin (Pannzer)	DNA_pol3_gamma3 superfamily		ND	
WP_002963780.1	216	Hypothetical protein	0.7	3.58	Not determined	No domain hit		ND	

## Data Availability

All the data used to support the findings of this study are included within the supplementary information file(s).

## Supplementary Materials

Supplementary 1 This table contains the strain name, genome accession numbers, and number of proteins of the 90 *Brucella* genomes used to conduct this study.

Supplementary 2 This Excel file contains the 177 surface-associated proteins resulted from our SCL prediction pipeline. The prediction results of 6 SCL tools used in the pipeline are shown.

Supplementary 3 This Excel file shows the 87 proteins: the first 38 proteins (with adhesion score ≥ 0.5) that are considered antigens with “high potential” and the rest 49 proteins (with adhesion score between 0.4 and 0.5) that are considered antigens with “intermediate potential.” The results of overall antigenicity and T- and B-cell epitope densities are also shown.

Supplementary 4 This Excel file contains the detailed calculation of the immunogenicity cumulative score that was derived from overall protein antigenicity, MHC-I density, MHC-II density, allele coverage, and B-cell density. In addition, it shows the results of conserved domain search and annotation results of the three tools: Blannotator, Pannzer, and eggNOG of the top 34 proteins.
