# Supplementary material for: Identification of Cross-Protective Potential Antigens against Pathogenic Brucella spp. through Combining Pan-Genome Analysis with Reverse Vaccinology
Source: J Immunol Res. 2018 Dec 9;2018:1474517. doi: 10.1155/2018/1474517 (PMC6304850; doi:10.1155/2018/1474517)
Supplement: Supplementary 1 — This table contains the strain name, genome accession numbers, and number of proteins of the 90 Brucella genomes used to conduct this study. [file 1474517.f1.pdf]

Organism/Name	Strain	Replicons	Proteins	No. of core Proteins (Intra)	No. of core Proteins (Inter)
Brucella melitensis bv. 1 str. 16M	16M	chromosome I:NC_003317.1/AE008917.1; chromosome II:NC_003318.1/AE008918.1	3099	2578	**1939**
Brucella melitensis ATCC 23457	ATCC 23457	chromosome I:NC_012441.1/CP001488.1; chromosome II:NC_012442.1/CP001489.1	3152		
Brucella melitensis M28	M28	chromosome 1:NC_017244.1/CP002459.1; chromosome 2:NC_017245.1/CP002460.1	3144		
Brucella melitensis M5-90	M5-90	chromosome I:NC_017246.1/CP001851.1; chromosome II:NC_017247.1/CP001852.1	3124		
Brucella melitensis NI	NI	chromosome I:NC_017248.1/CP002931.1; chromosome II:NC_017283.1/CP002932.1	3115		
Brucella melitensis bv. 3 str. Ether	ether	chromosome 1:NZ_CP007760.1/CP007760.1; chromosome 2:NZ_CP007761.1/CP007761.1	3130		
Brucella melitensis bv. 1 str. 16M	16M	chromosome 1:NZ_CP007763.1/CP007763.1; chromosome 2:NZ_CP007762.1/CP007762.1	3134		
Brucella melitensis	C-573	chromosome I:NZ_CP019679.1/CP019679.1; chromosome II:NZ_CP019680.1/CP019680.1	3099		
Brucella melitensis	20236	chromosome 1:NZ_CP008750.1/CP008750.1; chromosome 2:NZ_CP008751.1/CP008751.1	3140		
Brucella melitensis	2008724259	chromosome 1:NZ_CP016983.1/CP016983.1;	3125		

		chromosome 2:NZ_CP016984.1/CP016984.1		
Brucella melitensis	BwIM_AFG_63	chromosome 1:NZ_CP018478.1/CP018478.1; chromosome 2:NZ_CP018479.1/CP018479.1	3148	
Brucella melitensis	BwIM_IRN_37	chromosome 1:NZ_CP018486.1/CP018486.1; chromosome 2:NZ_CP018487.1/CP018487.1	3149	
Brucella melitensis	BwIM_IRQ_32	chromosome 1:NZ_CP018490.1/CP018490.1; chromosome 2:NZ_CP018491.1/CP018491.1	3145	
Brucella melitensis	BwIM_ITA_45	chromosome 1:NZ_CP018494.1/CP018494.1; chromosome 2:NZ_CP018495.1/CP018495.1	3131	
Brucella melitensis	BwIM_ITA_55	chromosome 1:NZ_CP018496.1/CP018496.1; chromosome 2:NZ_CP018497.1/CP018497.1	3126	
Brucella melitensis	BwIM_SYR_04	chromosome 1:NZ_CP018512.1/CP018512.1; chromosome 2:NZ_CP018513.1/CP018513.1	3149	
Brucella melitensis	BwIM_TKM_56	chromosome 1:NZ_CP018536.1/CP018536.1; chromosome 2:NZ_CP018537.1/CP018537.1	3148	
Brucella melitensis	BwIM_TUR_0	chromosome	3145	

	3	1:NZ_CP018540.1/CP018540.1; chromosome 2:NZ_CP018541.1/CP018541.1		
Brucella melitensis	BwIM_TUR_17	chromosome 1:NZ_CP018544.1/CP018544.1; chromosome 2:NZ_CP018545.1/CP018545.1	3146	
Brucella melitensis	BwIM_TUR_19	chromosome 1:NZ_CP018546.1/CP018546.1; chromosome 2:NZ_CP018547.1/CP018547.1	3149	
Brucella melitensis	BwIM_TUR_59	chromosome 1:NZ_CP018560.1/CP018560.1; chromosome 2:NZ_CP018561.1/CP018561.1	3147	
Brucella melitensis	BwIM_SAU_09	chromosome 1:NZ_CP018504.1/CP018504.1; chromosome 2:NZ_CP018505.1/CP018505.1	3146	
Brucella melitensis	BwIM_SYR_26	chromosome 1:NZ_CP018526.1/CP018526.1; chromosome 2:NZ_CP018527.1/CP018527.1	3145	
Brucella melitensis	BwIM_TUR_39	chromosome 1:NZ_CP018554.1/CP018554.1; chromosome 2:NZ_CP018555.1/CP018555.1	3144	
Brucella melitensis	QY1	chromosome I:NZ_CP022204.1/CP022204.1; chromosome II:NZ_CP022205.1/CP022205.1	3058	

Brucella melitensis	BY38	chromosome 1:NZ_CP022827.1/CP022827.1; chromosome 2:NZ_CP022828.1/CP022828.1	3141	
Brucella melitensis	BL	chromosome 1:NZ_CP022875.1/CP022875.1; chromosome 2:NZ_CP022876.1/CP022876.1	3146	
Brucella melitensis	QH61	chromosome 1:NZ_CP024653.1/CP024653.1; chromosome 2:NZ_CP024654.1/CP024654.1	3131	
Brucella melitensis	CIIMS-BH-2	chromosome I:NZ_CP025680.1/CP025680.1; chromosome II:NZ_CP025681.1/CP025681.1	3130	
Brucella melitensis	B3	chromosome 1:NZ_CP026005.1/CP026005.1; chromosome 2:NZ_CP026006.1/CP026006.1	3117	
Brucella melitensis	Rev.1 (passage 101)	chromosome I:NZ_CP024715.1/CP024715.1; chromosome II:NZ_CP024716.1/CP024716.1	3111	
Brucella melitensis	1	chromosome 1:NZ_LT962928.1/LT962928.1; chromosome 2:NZ_LT962929.1/LT962929.1	3120	
Brucella melitensis	1	chromosome 1:NZ_LT963350.1/LT963350.1; chromosome	3112	

		2:NZ_LT963351.1/LT963351.1		
Brucella melitensis	1	chromosome 1:NZ_LT962945.1/LT962945.1; chromosome 2:NZ_LT962946.1/LT962946.1	3120	
Brucella melitensis	1	chromosome 1:NZ_LT962940.1/LT962940.1; chromosome 2:NZ_LT962941.1/LT962941.1	3117	
Brucella melitensis	1	chromosome 1:NZ_LT962947.1/LT962947.1; chromosome 2:NZ_LT962948.1/LT962948.1	3104	
Brucella melitensis	1	chromosome 1:NZ_LT962910.1/LT962910.1; chromosome 2:NZ_LT962911.1/LT962911.1	3124	
Brucella melitensis	1	chromosome 1:NZ_LT962943.1/LT962943.1; chromosome 2:NZ_LT962944.1/LT962944.1	3119	
Brucella melitensis	1	chromosome 1:NZ_LT962914.1/LT962914.1; chromosome 2:NZ_LT962915.1/LT962915.1	3123	
Brucella melitensis	1	chromosome 1:NZ_LT962926.1/LT962926.1; chromosome 2:NZ_LT962927.1/LT962927.1	3122	
Brucella melitensis	1	chromosome 1:NZ_LT962922.1/LT962922.1;	3103	

		chromosome 2:NZ_LT962923.1/LT962923.1			
Brucella melitensis	1	chromosome 1:NZ_LT962916.1/LT962916.1; chromosome 2:NZ_LT962917.1/LT962917.1	3123		
Brucella melitensis	1	chromosome 1:NZ_LT962924.1/LT962924.1; chromosome 2:NZ_LT962925.1/LT962925.1	3121		
Brucella melitensis	1	chromosome 1:NZ_LT962912.1/LT962912.1; chromosome 2:NZ_LT962913.1/LT962913.1	3125		
Brucella melitensis	1	chromosome 1:NZ_LT962920.1/LT962920.1; chromosome 2:NZ_LT962921.1/LT962921.1	3116		
Brucella melitensis	1	chromosome 1:NZ_LT962918.1/LT962918.1; chromosome 2:NZ_LT962919.1/LT962919.1	3106		
Brucella melitensis	1	chromosome 1:NZ_LT963348.1/LT963348.1; chromosome 2:NZ_LT963349.1/LT963349.1	3108		
Brucella melitensis	1	chromosome 1:NZ_LT962930.1/LT962930.1; chromosome 2:NZ_LT962931.1/LT962931.1	3115		
Brucella melitensis	1	chromosome	3120		

		1:NZ_LT962932.1/LT962932.1; chromosome 2:NZ_LT962933.1/LT962933.1			
Brucella melitensis	1	chromosome 1:NZ_LT962938.1/LT962938.1; chromosome 2:NZ_LT962939.1/LT962939.1	3103		
Brucella melitensis	1	chromosome 1:NZ_LT962934.1/LT962934.1; chromosome 2:NZ_LT962935.1/LT962935.1	3126		
Brucella melitensis	1	chromosome 1:NZ_LT962936.1/LT962936.1; chromosome 2:NZ_LT962937.1/LT962937.1	3117		
Brucella melitensis	1	chromosome 1:NZ_LT962953.1/LT962953.1; chromosome 2:NZ_LT962954.1/LT962954.1	3121		
Brucella melitensis	1	chromosome 1:NZ_LT962951.1/LT962951.1; chromosome 2:NZ_LT962952.1/LT962952.1	3109		
Brucella melitensis	1	chromosome 1:NZ_LT962949.1/LT962949.1; chromosome 2:NZ_LT962950.1/LT962950.1	3100		
Brucella abortus bv. 1 str. 9-941	9-941	chromosome I:NC_006932.1/AE017223.1; chromosome II:NC_006933.1/AE017224.1	3153	2840	
Brucella abortus S19	S19	chromosome 1:NC_010742.1/CP000887.1; chromosome 2:NC_010740.1/CP000888.1	3151		

Brucella abortus 2308	2308	chromosome I:NC_007618.1/AM040264.1; chromosome II:NC_007624.1/AM040265.1	3153	
Brucella abortus A13334	A13334	chromosome 1:NC_016795.1/CP003176.1; chromosome 2:NC_016777.1/CP003177.1	3168	
Brucella abortus	BDW	chromosome 1:NZ_CP007681.1/CP007681.1; chromosome 2:NZ_CP007680.1/CP007680.1	3171	
Brucella abortus	BER	chromosome 1:NZ_CP007682.1/CP007682.1; chromosome 2:NZ_CP007683.1/CP007683.1	3130	
Brucella abortus	NCTC 10505	chromosome 1:NZ_CP007700.1/CP007700.1; chromosome 2:NZ_CP007701.1/CP007701.1	3140	
Brucella abortus bv. 9 str. C68	C68	chromosome 1:NZ_CP007705.1/CP007705.1; chromosome 2:NZ_CP007706.1/CP007706.1	3150	
Brucella abortus bv. 6 str. 870	870	chromosome 1:NZ_CP007709.1/CP007709.1; chromosome 2:NZ_CP007710.1/CP007710.1	3142	
Brucella abortus	63 75	chromosome 1:NZ_CP007663.1/CP007663.1; chromosome 2:NZ_CP007662.1/CP007662.1	3147	
Brucella abortus	BFY	chromosome 1:NZ_CP007738.1/CP007738.1; chromosome	3127	

		2:NZ_CP007737.1/CP007737.1			
Brucella abortus bv. 2 str. 86/8/59	86/8/59	chromosome 1:NZ_CP007765.1/CP007765.1; chromosome 2:NZ_CP007764.1/CP007764.1	3159		
Brucella abortus	BAB8416	chromosome 1:NZ_CP008774.1/CP008774.1; chromosome 2:NZ_CP008775.1/CP008775.1	3121		
Brucella abortus 104M	104M	chromosome 1:NZ_CP009625.1/CP009625.1; chromosome 2:NZ_CP009626.1/CP009626.1	3152		
Brucella abortus	BD	chromosome 1:NZ_CP022877.1/CP022877.1; chromosome 2:NZ_CP022878.1/CP022878.1	3115		
Brucella abortus	MC	chromosome 1:NZ_CP022879.1/CP022879.1; chromosome 2:NZ_CP022880.1/CP022880.1	3109		
Brucella abortus	B4	chromosome I:NZ_CP025743.1/CP025743.1; chromosome II:NZ_CP025744.1/CP025744.1	3123		
Brucella suis 1330	1330	chromosome I:NC_004310.3/AE014291.4; chromosome II:NC_004311.2/AE014292.2	3161	2484	
Brucella suis ATCC 23445	ATCC 23445	chromosome I:NC_010169.1/CP000911.1; chromosome II:NC_010167.1/CP000912.1	3158	2484	
Brucella suis 1330	1330	chromosome I:NC_017251.1/CP002997.1; chromosome II:NC_017250.1/CP002998.1	3164	2484	

Brucella suis VBI22	VBI22	chromosome I:NC_016797.1/CP003128.1; chromosome II:NC_016775.1/CP003129.1	3167	2484		
Brucella suis bv. 1 str. S2	S2	chromosome I:NZ_CP006961.1/CP006961.1; chromosome II:NZ_CP006962.1/CP006962.1	3167	2484		
Brucella suis bv. 2	PT09143	chromosome I:NZ_CP007691.1/CP007691.1; chromosome II:NZ_CP007692.1/CP007692.1	3162	2484		
Brucella suis bv. 2	PT09172	chromosome I:NZ_CP007693.1/CP007693.1; chromosome II:NZ_CP007694.1/CP007694.1	3162	2484		
Brucella suis bv. 2	Bs364CITA	chromosome I:NZ_CP007697.1/CP007697.1; chromosome II:NZ_CP007698.1/CP007698.1	3163	2484		
Brucella suis bv. 2	Bs396CITA	chromosome I:NZ_CP007720.1/CP007720.1; chromosome II:NZ_CP007721.1/CP007721.1	3164	2484		
Brucella suis bv. 2	Bs143CITA	chromosome I:NZ_CP007695.1/CP007695.1; chromosome II:NZ_CP007696.1/CP007696.1	3162	2484		
Brucella suis	513UK	chromosome 1:NZ_CP007717.1/CP007717.1; chromosome 2:NZ_CP007716.1/CP007716.1	3165	2484		
Brucella suis bv. 3 str. 686	686	chromosome	3121	2484		

		1:NZ_CP007719.1/CP007719.1; chromosome 2:NZ_CP007718.1/CP007718.1			
Brucella suis	BSP	chromosome 1:NZ_CP008757.1/CP008757.1; chromosome 2:NZ_CP008756.1/CP008756.1	3153	2484	
Brucella suis	Human/AR/US/1981	chromosome I:NZ_CP010850.1/CP010850.1; chromosome II:NZ_CP010851.1/CP010851.1	3164	2484	
Brucella suis	2004000577	chromosome 1:NZ_CP016981.1/CP016981.1; chromosome 2:NZ_CP016982.1/CP016982.1	3154	2484	
Brucella suis	QH05	chromosome 1:NZ_CP024420.1/CP024420.1; chromosome 2:NZ_CP024421.1/CP024421.1	3151	2484	
Brucella suis	ZW046	chromosome 1:CP009096.1; chromosome 2:CP009097.1	3124	2484	
Brucella suis	ZW043	chromosome 1:CP009094.1; chromosome 2:CP009095.1	3090	2484	
